# Downhill seed dispersal by temperate mammals: a potential threat to plant escape from global warming

**DOI:** 10.1038/s41598-019-51376-6

**Published:** 2019-10-17

**Authors:** Shoji Naoe, Ichiro Tayasu, Yoichiro Sakai, Takashi Masaki, Kazuki Kobayashi, Akiko Nakajima, Yoshikazu Sato, Koji Yamazaki, Hiroki Kiyokawa, Shinsuke Koike

**Affiliations:** 1 Forestry and Forest Products Research Institute, Matsunosato 1, Tsukuba, Ibaraki 305–8687 Japan; 2 Research Institute for Humanity and Nature, 457-4 Motoyama, Kamigamo, Kita-ku, Kyoto, 603-8047 Japan; 3 Center for Ecological Research, Kyoto University, Hirano 2-509-3, Otsu, Shiga, 520-2113 Japan; 4 Lake Biwa Environmental Research Institute, 5-34 Yanagasaki, Ohtsu, Shiga, 520-0022 Japan; 5 College of Bioresource Sciences, Nihon University, Fujisawa, Kanagawa 252-8510 Japan; 6 Ibaraki Nature Museum, 700 Ohsaki, Bando, Ibaraki 306-0622 Japan; 7 Laboratory of Biodiversity Science, School of Agriculture and Life Sciences, University of Tokyo, 1-1-1 Yayoi, Bunkyo-ku, Tokyo, 113-8656 Japan; 8 Tokyo University of Agriculture and Technology, 3-5-8 Saiwai, Fuchu, Tokyo, 183-8509 Japan; 9 Present Address: Tohoku Research Center, Forestry and Forest Products Research Institute, 92-25 Nabeyashiki, Shimokuriyagawa, Morioka, Iwate, 020–0123 Japan; 10 Present Address: Rakuno Gakuen University, 582 Bunkyodai-Midorimachi, Ebetsu, Hokkaido, 069-8501 Japan; 11 Present Address: Department of Forest Science, Tokyo University of Agriculture, 1-1-1 Sakuragaoka, Setagaya, Tokyo, 156-8502 Japan

**Keywords:** Ecology, Climate-change ecology, Plant ecology, Forest ecology

## Abstract

Vertical seed dispersal, i.e. seed dispersal towards a higher or lower altitude, is considered a critical process for plant escape from climate change. However, studies exploring vertical seed dispersal are scarce, and thus, its direction, frequency, and mechanisms are little known. In the temperate zone, evaluating vertical seed dispersal of animal-dispersed plants fruiting in autumn and/or winter is essential considering the dominance of such plants in temperate forests. We hypothesized that their seeds are dispersed towards lower altitudes because of the downhill movement of frugivorous animals following the autumn-to-winter phenology of their food plants which proceeds from the mountain tops to the foot in the temperate zone. We evaluated the vertical seed dispersal of the autumn-fruiting wild kiwi, *Actinidia arguta*, which is dispersed by temperate mammals. We collected dispersed seeds from mammal faeces in the Kanto Mountains of central Japan and estimated the distance of vertical seed dispersal using the oxygen isotope ratios of the dispersed seeds. We found the intensive downhill seed dispersal of wild kiwi by all seed dispersers, except the raccoon dog (bear: mean −393.1 m; marten: −245.3 m; macaque: −98.5 m; and raccoon dog: +4.5 m). Mammals with larger home ranges dispersed seeds longer towards the foot of the mountains. Furthermore, we found that seeds produced at higher altitudes were dispersed a greater distance towards the foot of the mountains. Altitudinal gradients in autumn-to-winter plant phenology and other mountain characteristics, i.e. larger surface areas and more attractive human crops at lower altitudes compared to higher altitudes, were considered drivers of downhill seed dispersal via animal movement. Strong downhill seed dispersal by mammals suggests that populations of autumn-to-winter fruiting plants dispersed by animals may not be able to sufficiently escape from current global warming in the temperate zone.

## Introduction

Global warming threatens the persistence of plants^1,2^. Recent plant range shifts towards the poles and higher altitudes^3,4^, higher plant mortality, decreased growth, and lower reproductive and recruitment rates near the warmer boundaries of plant ranges^5,6^ indicate that suitable plant habitats are being shifted towards higher latitude or altitude areas. As plants cannot move themselves, they must locally adapt to warmer environments or disperse their seeds towards colder areas^7^.

Seed dispersal towards a higher or lower altitude (i.e. vertical seed dispersal), is probably a key characteristic of plants that enables plant populations to escape from increasing temperatures^8,9^, considering that the temperature decrease with increasing altitude (ca. −0.6 °C per 100 m altitude) is 100–1000 times more than that with an equivalent latitudinal distance^10^. Actually, plant geographical distributions are tracking global warming altitudinally rather than latitudinally, and the extent of tracking is considered to be large in plants with better-dispersed traits^7^. Nevertheless, studies related to vertical seed dispersal are very limited: exceptions are studies which confirm that juvenile trees above alpine treelines are from seed sources below treelines by conducting genetic parentage analysis between juvenile and adult trees^11,12^. Remarkably, vertical seed dispersal itself was only recently evaluated, as it was too costly and too technically difficult^13–15^. There are only two recent studies which evaluated vertical seed dispersal: one by using the oxygen isotope ratios of seeds^8^, and another by using experimental seed mimics^9^. The lack of information on vertical seed dispersal, such as its direction, frequency, and mechanism, limits our understanding of how plants and forests respond to global warming.

**Figure 1:**
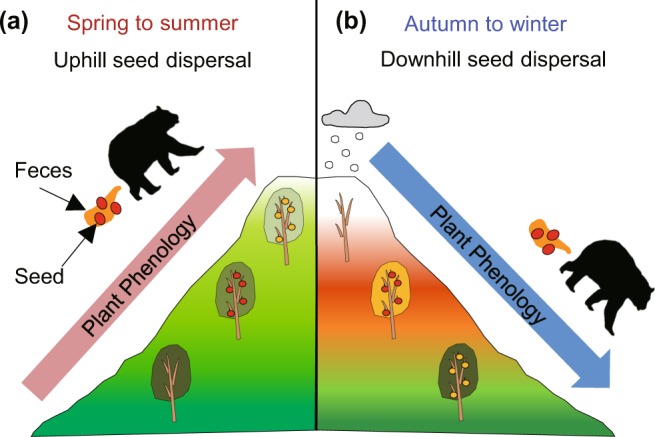
Hypothetical relationship between vertical seed dispersal and fruiting season. (**a**) Vertical seed dispersal toward the mountain tops by mammals that are following the spring-to-summer plant phenology^8^. The spring-to-summer plant phenology proceeds from the foot to the mountaintops. Midway through the season, fruits are no longer available at low altitudes, ripe fruits are available at middle altitudes, and fruits are not yet ripe at high altitudes. (**b**) Hypothetical vertical seed dispersal toward the foot of the mountains by mammals that are following the autumn-to-winter plant phenology. The autumn-to-winter plant phenology proceeds from the top to the foot of mountains. Midway through the season, fruits are no longer available at high altitudes, ripe fruits are available at middle altitudes, and fruits are not yet ripe at low altitudes.

Naoe *et al*. developed a method to detect vertical seed dispersal itself, using the altitudinal gradient of the oxygen isotope ratios of seeds to locate the altitudes of mother plants^8,13^. They targeted summer-fruiting wild cherries in temperate forests in Japan and showed that their seeds are dispersed towards the mountain tops by frugivorous mammals, thereby successfully escaping global warming. They concluded that this biased seed dispersal was due to the ascent of mammals following the spring-to-summer phenology of their food plants, proceeding from the foot to the tops of mountains in the temperate zone^16,17^ (Fig. 1a). These data suggest that spring- and summer-fruiting plants dispersed by animals have a possibility to escape global warming. However, this may not be the case with many other plants which fruit later.

Seed dispersal towards lower altitudes can occur if frugivorous animals descend following autumn-to-winter plant phenology in the temperate zone, proceeding from the top to the foot of mountains^8,16,18^ (Fig. 1b). In fact, many temperate frugivorous birds and mammals are known to descend the mountains from autumn to winter^19–22^. We hypothesized that the seeds of autumn- and winter-fruiting plants are dispersed towards lower altitudes because of the downhill movement of frugivorous animals following the autumn-to-winter phenology of their food plants. Considering that seed dispersal towards lower altitudes under global warming can lead to plant population reductions because reduced plant performance is expected at lower altitudes due to competition with more warm-adapted plants^23–25^ and that many of temperate woody species depend on animals for seed dispersal and produce fruits in autumn and winter^26^, testing this hypothesis is essential from both a biodiversity conservation and ecosystem functioning of temperate forests perspective.

**Figure 2:**
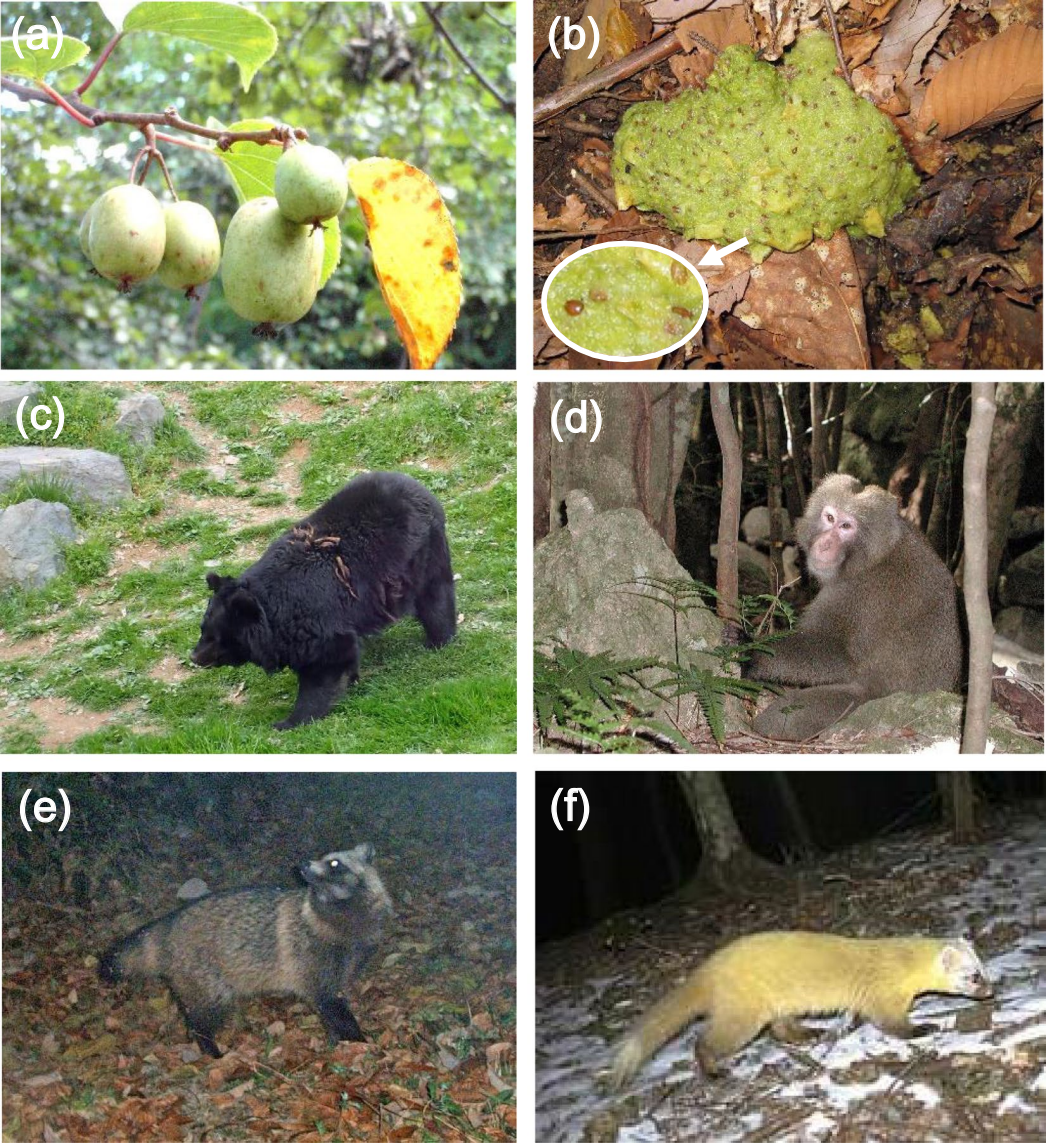
Wild kiwi *Actinidia arguta* and its seed dispersers. (**a**) Fruits of wild kiwi, (**b**) Faeces of Asian black bears containing seeds of wild kiwi, (**c**) Asian black bear, (**d**) Japanese macaque, (**e**) Raccoon dog, and (**f**) Japanese marten.

To test the hypothesis, we evaluated the vertical seed dispersal of the autumn-fruiting wild kiwi *Actinidia arguta*, which is dispersed by temperate mammals^27,28^ (Fig. 2). We collected dispersed seeds from mammal faeces in central Japan and estimated the vertical seed dispersal distance using the oxygen isotope ratios of dispersed seeds.

## Results

**Figure 3:**
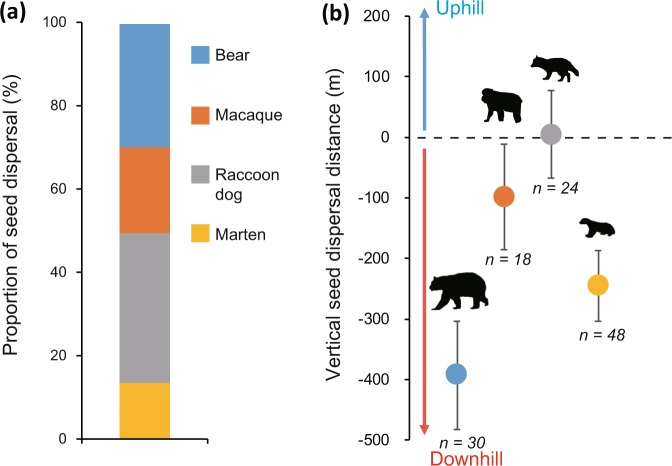
Downhill seed dispersal by mammals. (**a**) Proportion of seed dispersal by Asian black bears, Japanese macaques, raccoon dogs, and Japanese martens. (**b**) Vertical seed dispersal distance by each mammal (mean ± standard error). Positive and negative values indicate seed dispersal toward the top and foot of the mountains, respectively.

A total of 15,661 dispersed *Actinidia arguta* seeds were collected from the faeces of the Asian black bear, the Japanese macaque, the raccoon dog, and the Japanese marten, listed in descending order of body size (See Fig. 2 and Table S1). Each mammal accounted for more than 10% of the total dispersed seeds (Fig. 3a). Mean oxygen isotope ratio of seeds collected in the feces of Asian black bears, Japanese macaques, raccoon dogs, and Japanese martens were 15.7 ± 0.2, 16.0 ± 0.2, 15.7 ± 0.1, and 15.4 ± 0.1 m SE, respectively. The overall range of the oxygen isotope ratio of dispersed seeds was 13.53–17.45‰. Because the range of oxygen isotope ratio of the calibration line (i.e., the negative correlation between altitudes and the oxygen isotope ratio of non-dispersed seeds collected at 600–1,280 m a.s.l., see *Materials and Methods*) was 15.09–17.73‰^13^, some vertical seed dispersal estimate is the result of an extrapolation (see *Discussion* for details).

**Figure 4:**
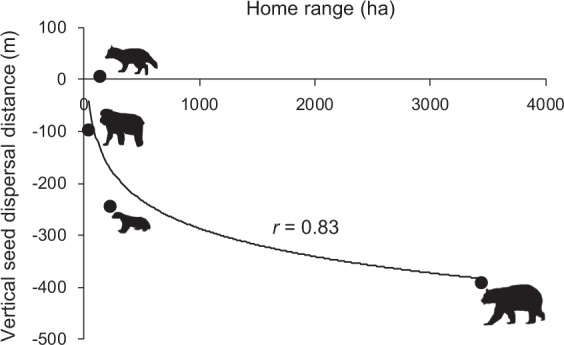
Mammals having larger home range sizes dispersed seeds longer toward the foot of the mountains. A logarithmic regression between mean vertical seed dispersal distance and home range size of each mammal was conducted: y = −78.07 ln(x) + 252.86, *r* = 0.83.

**Figure 5:**
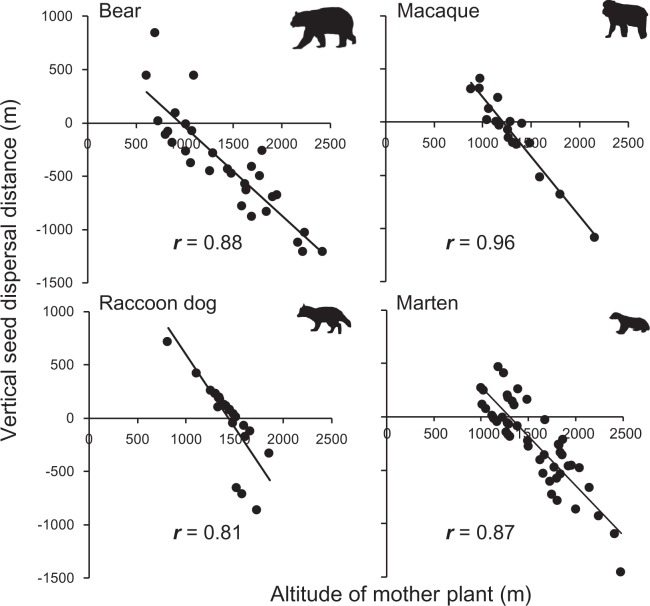
Seeds produced at higher altitudes were dispersed longer toward the foot of the mountains. Linear regressions between vertical seed dispersal distance and altitude of the mother plant were conducted (Asian black bears, *r* = 0.88, *t* = −9.78, *P* < 0.0001, *n* = 30; Japa*n*ese macaques, *r* = 0.96, *t* = −13.56, *P* < 0.0001, *n* = 18; raccoon dogs, *r* = 0.81, *t* = −6.51, *P* < 0.0001, *n* = 24; Japanese martens, *r* = 0.87, *t* = −12.07, *P* < 0.0001, *n* = 48).

Based on the Bayesian inference, the direction of seed dispersal by all mammal species was biased towards the foot of the mountains (Asian black bear: 63% of the seeds were dispersed downhill—the 95% credible intervals of its dispersal distance did not cross zero, and 10% of them were dispersed uphill, and the remaining 27% were neither, Japanese macaque: 28% downhill, 17% uphill and 55% neither, raccoon dog: 13% downhill, 8% uphill and 79% neither, and Japanese marten: 40% downhill, 8% uphill and 52% neither)(Fig. S2). Mean vertical seed dispersal distances by Asian black bears, Japanese macaques, raccoon dogs, and Japanese martens were −393.1 ± 88.1, −98.5 ± 84.8, + 4.5 ± 70.7, and −245.3 ± 57.5 m SE, respectively (Figs 3b and S3). The dispersal distances differed because mammals with larger home ranges dispersed seeds further towards the foot of the mountains (Fig. 4). Vertical seed dispersal distance differed among mother plants at different altitudes (Fig. 5). Seeds produced by mother plants at high altitudes were intensively dispersed towards the foot of the mountains, while seeds produced at lower altitudes were moderately dispersed towards mountain tops.

## Discussion

Highly biased seed dispersal towards the foot of the mountains, coinciding with the known descent of temperate frugivores including our target mammals during the autumn-to-winter season^19^, was probably due to the autumn-to-winter phenology of the food plants of mammals (Fig. 1). The autumn-to-winter phenology of plants, including fruit production, proceeds from the top to the foot of mountains in the temperate zone^16,18,29,30^. Furthermore, temperate mammals and birds are highly dependent on plants during autumn and winter, when fruits are abundant^31–33^. Thus, mammals are likely to descend mountains following the phenology of their food plants, resulting in biased seed dispersal towards the foot of the mountains (Fig. 1b). However, mammals’ simple tracking of descending plant phenology does not seem to be the sole causal factor. This is because seed retention time in the gut of target mammals does not exceed 45 hours^34^, which is not long enough for plant phenology to descend 300 m, the distance achieved by seed dispersal. In addition to plant phenology, two causal factors, which are not mutually exclusive, are presumed for enhancement of downhill seed dispersal. These factors are the topography of mountains and daily foraging patterns of mammals. Larger surface areas at lower altitudes than at higher altitudes can cause downhill seed dispersal if mammals move randomly in mountains^9^, although animal behaviour can mask this bias depending on the situation (i.e. strong uphill seed dispersal by mammals in summer-fruiting cherries)^8^. In addition, although target mammals inhabit deciduous forests, some individuals routinely visit human settlements to feed on agricultural crops and then return to the forests^19,35^. This foraging pattern can result in downhill seed dispersal because compared to deciduous forests, human settlements are usually located at lower altitudes. In fact, bears in the study site frequently consumed autumn crops, including walnuts (*Juglans mandshurica*), chestnuts (*Castanea crenata*), and persimmons (*Diospyros kaki*) in abandoned fields near human settlements located 500–900 m a.s.l., as well as acorns of the Mongolian oak (*Quercus crispula*), which is distributed in the deciduous forests above 800 m a.s.l^35^. Although mother plants located at low altitudes recorded several uphill seed dispersal, possibly because of the ascent of bears from human settlements, the frequency was lower compared with that of downhill seed dispersal of mother plants at higher altitudes (Fig. 5). Owing to the fact that some or all fruits available near human settlements are preferred by all target mammals^19,36^, the same mechanism would have occurred with other mammals. It is noteworthy that the triggers of downhill seed dispersal (i.e. larger surface areas and attractive human crops at low altitudes) are very common phenomena worldwide^37^, and thus, likely to cause downhill dispersal of autumn-to-winter fruiting plants in other temperate mountain systems.

By targeting fleshy-fruited plants of which seeds are dispersed via animal feces, we supported our hypothesis that the seeds of autumn-fruiting plants are dispersed towards the foot of mountains by animals though we could not quantify the independent effect of the three triggers causing downhill seed dispersal. Downhill seed dispersal can lead to a reduction in the plant population under global warming, because plant performance (i.e., survival, growth, and reproduction) is strongly reduced by novel competitive plants in lower altitude environments^23,25^. In fact, a study^38^ reported lower fruit production of *Actinidia arguta* in lower altitudes compared to the centre of vertical species distribution. In our study, we observed a low but non-negligible frequency of uphill seed dispersal, which can help plant escape from warming temperatures (Figs 5, S2 and S3). Does such uphill seed dispersal substantially contribute to maintenance of the dominance of autumn-to-winter fruiting plants in future plant communities? Strong uphill seed dispersal is expected to occur in spring-to-summer fruiting plants dispersed by animals^8^ and relatively unbiased, wind- and explosion-mediated seed dispersal is likely to occur in abiotic-dispersed plants. Thus, in terms of dispersal, the frequency of uphill seed dispersal of autumn-to-winter fruiting plants will be low compared to other plants, and it make them difficult to maintain the current dominance at higher altitudes. But the likelihood that uphill seed dispersal events will translate into the uphill movement of a plant population depends on both the total number of seeds dispersed and the proportion of these that survive to reproduce^7^. The proportion of recruitment depends on environments which can be very different among locations. For example, in East Asia, where plant species richness is high^39^, interspecific competition for recruitment is intense compared to Europe^40^ and thus much uphill seed dispersal will be needed. In mountaintops, smaller land surface area is likely to increase interspecific competition, and harsh environments such as strong wind and poor soil^41^ will prevent the colonization of stress-intolerant plants coming from lower altitudes. Therefore, we need to carefully evaluate the recruitment performance of focal plants to answer whether they can escape from warming temperature by the aid of observed uphill seed dispersal. As for our target *A. arguta*, its recruitment performance is quite low compared to that of many other co-occurring woody species^*unpubl*^ and thus maintaining its current dominance in plant communities seems difficult. It is noteworthy that we observed the considerable amount of dispersed seeds were neither of downhill or uphill dispersal in terms of the 95% credible intervals (Fig. S2). These relatively short distance seed dispersal can contribute to maintain plant population at its present location until the location becomes completely unsuitable for recruitment and thus may slow total population decrease in mountains. In addition to uphill seed dispersal, rapid evolution *in situ* might be how plants cope with warming crises, but the evidence from the paleoecological record of range shifts and local extinctions does not support this possibility^7^. Importantly, rapid evolution is not expected to occur in woody species which have long generation times^42^ and thus need a longer period of time to adapt. In this study, we focused on fleshy-fruited plants, one of three types of animal-dispersed plants (the others are synzoochorous and epizoochorous plants). Fleshy-fruited plants account for ca. 35–42% of woody species in temperate forests^43^ and that most of them fruit in the autumn-to-winter period. Therefore, aside from recruitment performance, dominance of downhill seed dispersal against uphill one observed in *A. arguta* possibly suggest large changes in species composition and ecological function in temperate forests due to global warming. The same phenomenon may occur in synzoochorous trees whose nuts are transported and cached by animals represented by rodents and corvids^44^. Rodents have small home ranges and disperse seeds short distances^45,46^ and thus they are unlikely to conduct detectable vertical seed dispersal. In addition, they mainly act as seed predators rather than seed dispersers^44^. On the other hand, corvids such as nutcrackers and jays have large home ranges and can disperse seeds long distances^47–49^, and are likely to conduct downhill seed dispersal in autumn and winter. If downhill seed dispersal frequently occurs in synzoochorous trees, the forest changes will be even more intense, because this plant group includes *Fagaceae* trees, which dominate in temperate forests in terms of biomass and produce acorns in autumn on which many animals depend. In the light of the precautionary approach, it is necessary to evaluate whether our hypothesis holds true in whole animal-dispersed plants in the temperate zone.

Although seed dispersal generally occurred in a downhill direction, seed dispersal varied in different animals. Mammals with larger home ranges dispersed seeds longer towards the foot of the mountains (Fig. 4). Macaques and raccoon dogs dispersed seeds less than 100 m on average but bears and martens dispersed seeds several hundred meters towards lower altitudes. Therefore, fruit consumption by macaques and raccoon dogs reduces the distance of downhill seed dispersal by decreasing the opportunity for consumption and dispersal of the same seeds by bears and martens. Seed dispersers with small home ranges do not contribute much to plant expansion but they can contribute to plant persistence at their present location. The directionality of vertical seed dispersal can affect plants positively or negatively under climate change: uphill or downhill seed dispersal are advantageous or disadvantageous, respectively, for plant escape and expansion under global warming, and are disadvantageous or advantageous, respectively, under global cooling. Considering that a bias in the direction of seed dispersal can be harmful or helpful to a plant population depending on the conditions, having multiple seed dispersers with different seed dispersal distances may help plants survive climate change in the long term.

It is noticeable that our evaluation on vertical seed dispersal includes some caveats. First, some vertical seed dispersal is the result of an extrapolation: the calibration line (see *Materials and Methods* for details) is based on non-dispersed seeds collected at the lower altitudes (i.e. 600–1,280 m a.s.l.) and the range of oxygen isotope ratio (15.09–17.73‰)^13^ was smaller than that of dispersed seeds (13.53–17.45‰). This indicates that its statistical reliability of estimate is relatively poor, and it was reflected in the large 95% credible interval of each seed dispersal distance, especially in long-distance seed dispersal (Fig. S2). We estimated several strong downhill seed dispersal exceeding 1,000 m vertical distance in terms of mean value. Although our study area is steep and thus vertical seed dispersal can occur with short animal movement^8^, mammals need to move more than 10,000 m horizontal distance to achieve 1,000 m vertical distance. Such long movement during gut passage time seems rare for target mammals except bears^34,50–52^, although the literature studying the movement pattern and gut passage time of target mammals are limited. Therefore, we need to be careful about the interpretation of the mean value of such extreme long-distance vertical seed dispersal. Although the accuracy of the estimate does not affect overall patterns (i.e., dominance of downhill seed dispersal detected in the 95% credible interval) (Fig. S2), in future study, it is desirable to find ways to increase the accuracy seed dispersal estimate including using a calibration line based on non-dispersed seeds collected in the full altitudinal range of focal plant species, and multiple isotopes for estimate^53^. Second, in this study, we only focused on seed dispersal process and did not investigate how target mammals treat seeds which can differ among species. Considering that the proportion of damaged seeds of *A. arguta* in feces is less than 7%, regardless of target mammals^28,54^, and that the proportion of seedling emergence of *Prunus* from faeces do not differ among target mammals^*unpub*^, differences in seed treatment among species would probably be small. Diploendozoochory (i.e., seed dispersal that involves the ingestion of the seed by two or more separate species of animals in sequence)^55^ by target mammals would probably be rare, because the frequency of their faeces containing the remains of frugivorous bird/mammal is nearly 0% in autumn^32,56,57^. Third, we could not evaluate annual variation of vertical seed dispersal. In the study year, fruit production of *Fagaceae* trees was relatively poor^35^. When fruit availability is low, frugivores spatially move longer searching for fruits and thus disperse seeds longer^34,58^. Therefore, the magnitude of vertical seed dispersal distance may decrease in abundant fruiting year. Masting of such dominant animal-dispersed plants may also affect seed dispersal patterns by changing the population and behaviour of various animals^31,59–61^.

Previous studies predicting vegetation dynamics under global warming have ignored or poorly incorporated the process of seed dispersal. However, such predictions can be quite different from actual vegetation dynamics^62,63^. Especially, predictions about vertical vegetation dynamics may be unrealistic because, as we showed in this and previous studies^8^, vertical seed dispersal can be highly directional. We know very little about vertical seed dispersal; however, we need to approach its mechanisms to better understand vegetation dynamics. In our study, the vertical seed dispersal distance was correlated with mammal home ranges (Fig. 4) and we found, opposite, but similar, absolute vertical seed dispersal distances between autumn-fruiting kiwis and summer-fruiting cherries (by bears: −393.1 m in kiwi and +307.2 m in cherry, by martens: −245.3 m in kiwi and +193.0 m in cherry)^8^. These data suggest that the distance and direction of vertical seed dispersal by animals in the temperate zone may be predicted by the home range of the seed disperser and the fruiting season of the plants. However, species-specific animal behaviour may also affect vertical seed dispersal patterns. A study^9^ reported less limited uphill seed dispersal towards deforested mountaintops by habitat generalist species (the red fox *Vulpes vulpes*) compared to the one by forest specialist species (the pine marten *Martes martes*). In our study, the mean vertical seed dispersal distance by raccoon dogs was larger than that expected from their home range (Fig. 4): they dispersed seeds uphill more frequently than that by other mammals (Figs S2 and S3). Raccoon dogs are known to communicate to each other by the communal utilization of latrines of which locations are fairly stable throughout the year^64^. The adherence to shared latrines at high altitudes, even after the environmental conditions such as fruit availability and weather, became harsh in autumn, might have caused them to ascend mountains resulting in uphill seed dispersal. We also need to consider the fact that vertical seed dispersal may be affected by the characteristic of each mountain system, such as steepness and vertical distribution of the habitat (especially in a fragmented landscape), which promote or inhibit animal movements, and summit altitude, which limits seed dispersal distance^9^. Moreover, global warming itself can change the behaviour and ecology of seed dispersers^65,66^, resulting in changes in vertical seed dispersal in the future. Changes in altitudinal vegetation due to seed dispersal by animals and climate change are also likely to alter the movement and ecology of animals (i.e. feedback of animal behaviour). Much more information about the response of animals and plants to global warming along an elevational gradient is needed for reliable prediction how global warming changes future vertical seed dispersal. In addition to studying vertical seed dispersal *per se*, linking knowledge of well-studied horizontal seed dispersal to vertical one will be effective, considering that both seed dispersal are different aspects of the same seed movement which is intrinsically described in three dimensions. In fact, a study^9^ reported a positive correlation between vertical and horizontal seed dispersal distance. Furthermore, a positive correlation between seed dispersal distance and the home range of animals was also reported in studies of horizontal seed dispersal^67,68^. Accumulating the studies of vertical seed dispersal and utilizing our knowledge of horizontal seed dispersal are both necessary to reveal the mechanisms of vertical seed dispersal. Clarifying the mechanisms of vertical seed dispersal and evaluating the recruitment performance of seeds dispersed to different altitudes, will enable realistic predictions of vegetation dynamics under global warming conditions.

## Material and Methods

### Overview of vertical seed dispersal estimation

We can evaluate vertical seed dispersal distance, that is, the vertical distance between the altitude of each dispersed seed and that of its mother plant estimated from the oxygen isotope ratio of the dispersed seed^8^. Negative correlations between altitudes and oxygen isotope ratio of seeds in various temperate plants, including the target species, *Actinidia arguta*, were previously reported^13^. We can determine the altitude of a mother plant by using the negative correlation and oxygen isotope ratio of a dispersed seed, and then estimate the vertical seed dispersal distance by subtracting the altitude of the mother plant from that of the dispersed seed^8,13^.

### Study sites

This study was conducted at Okutama in the Kanto Mountains (ca. 8,000 km^2^), approximately 100 km west of Tokyo, Japan (See Fig. S1). The mean annual precipitation from 1981 to 2010 at 530 m above sea level (a.s.l.) was 1,624 mm, and the mean annual temperature was 11.9 °C (range: 1.3 °C in January to 23.2 °C in August)^69^. The study area is mountainous and mostly covered with forest vegetation. Natural forests and conifer plantations (*Cryptomeria japonica* or *Chamaecyparis obtusa*) cover 41.3% and 50.3% of the area, respectively^28^. The natural forests are dominated by *Quercus serrata* and *Castanea crenata* in the hilly zone (400–800 m a.s.l.); *Quercus crispula* and *Fagus crenata* in the montane zone (800–1800 m a.s.l.); and *Abies veitchii* and *Tsuga diversifolia* in the subalpine zone (1800–2500 m a.s.l.)^28,35,70–72^. Forest floors are densely covered by herbs and shrubs, represented by a dwarf bamboo *Sasamorpha borealis*, but the drastic increase of the Sika deer (*Cervus nippon*) population since the 1990s is now threating the understory vegetation^70^. Most human settlements in the area are found between 500 and 900 m a.s.l^35^.

### Selection of plant species

We selected *A. arguta* as the study species, which is widely distributed in Japan, Korea, Northern China, and Russian Siberia. The vertical distribution of *A. arguta* in the study region is from the sea coast to the subalpine zone, but its main distribution is the montane zone (i.e., 800–1800 m a.s.l. at study site)^73^. *Actinidia arguta* is a deciduous woody vine with a mean diameter at breast height (DBH) of 4.4 cm^74^. *A. arguta* is a stem twiner^74^ and it prefers light habitats represented by forest edges and gaps^75^. It produces oval green fruits (average 19 mm in length and 17.5 mm in width) from mid-August to November^27^. Each fruit contains ca. 80 seeds (2 mm diameter). Inconspicuously coloured fruits, large fruit sizes, and many small seeds in a fruit are characteristics of mammal-dispersed plants^26^. In fact, *A. arguta* is one of the most preferred fruits of temperate frugivorous mammals^76^ and is seldom eaten by birds^27^. At study site, *A. arguta* is the most preferred fruits of mammals in terms of the percent of frequency of occurrence in the faeces and the number of dispersed seeds^*unpub*^.

### Survey route

We established a 16 km survey route (550–1,650 m a.s.l.) to collect mammal-dispersed seeds along unpaved forestry roads and animal trails (0.8–2 m in width). The route includes all representative vegetation types in the study site^77^. Asian black bears (*Ursus thibetanus*, frugivorous), wild boars (*Sus scrofa*, not frugivorous*)*, sika deer (*Cervus Nippon*, not frugivorous), Japanese serows (*Capricornis crispus*, not frugivorous), Japanese macaques (*Macaca fuscata*, frugivorous), badgers (*Meles meles*, frugivorous), raccoon dogs (*Nyctereutes procyonoides*, frugivorous), and Japanese martens (*Martes melampus*, frugivorous) in descending order of body size, were observed on the route using four camera traps set in 2010 and 2011^8^. As for Asian black bears, which had the largest home ranges among the targeted mammals (i.e. a mean of 3,450 ha in the study area), seven individuals were captured and tagged in 2010 and 2011 around the route^35^. These indicate that the mammalian biota is rich and all representative frugivorous mammals in Central Japan inhabit the region around the study route^19,36^.

### Collection of mammal-dispersed seeds

To collect dispersed seeds in mammalian faeces, we visited the 16 km survey route at 10-day intervals from July to October in 2010 and July to November in 2011 (accumulated sampling distance: 448 km in total in two years). All fresh faeces found on the route were examined *in situ* for *A. argut*a seeds. Mammal species were identified based on the shape and smell of the faeces, according to a reference^28^. Each faecal sample was washed through a series of sieves (2.0, 1.0, and 0.5 mm mesh), after which the number of mature intact seeds was counted. Collected and counted seeds from bear faeces were accidentally lost during transportation for subsequent isotope analysis. However, we were able to use seeds from bear faeces along or near the route collected in 2011 for a different study^35^ for isotope analyses. The bear faeces were efficiently collected by visiting places where GPS-attached bears inhabited from late May to November^35^. We picked up *A. arguta* seeds from the faeces. Approximately, 40% of the seeds were from faeces collected along the route, and the remaining 60% were from faeces collected within a horizontal distance of 800 m from the route. The resulting altitudinal locations of the faeces were 690–1,540 m a.s.l. which was within the altitudinal range of the survey route (i.e. 550–1,650 m a.s.l.). Considering that the sampling locations of our study and that of the previous study^35^ were almost identical, we assumed that the use of the replacement bear faeces would not affect our results.

### Isotope analysis

We subjected three seeds per faecal sample to the oxygen isotope analysis^8^. The seed coat was removed from each seed and ground to powder^78^. Each seed coat was weighed (approximately 0.15 mg) into silver capsules (Säntis Analytical, Milan, Italy) and rolled into balls for continuous flow (CF) combustion and isotope ratio mass spectrometry (IRMS) analysis using a high-temperature elemental analyser (TC/EA, Thermo Fisher Scientific, Waltham, Massachusetts, USA) coupled online with a mass spectrometer (Delta V plus; Thermo Fisher Scientific) using a ConFlo IV interface (Thermo Fisher Scientific). The molecular water absorbed from the atmosphere was removed in advance by vacuum-drying the samples for at least 1 night before measurements to avoid water contamination in the samples. The oxygen isotope analyses were performed by measuring the CO obtained by high-temperature carbothermic reduction of the endocarp (1,400 °C) in the presence of an excess of carbon by means of a TC/EA. Before entering the mass spectrometer, the helium stream containing CO was separated from H_2_ and N_2_ by a 1.4 m molecular sieve chromatographic column maintained at 90 °C. The sample gases were calibrated by measuring reference substances (IAEA-601 and IAEA-602 benzoin acid; 23.14‰ and 71.28‰, respectively) of known isotope composition^79^.

The isotopic composition of a sample is conventionally expressed as the ‘*δ*’ value by comparison with the international primary reference material (V-SMOW) as follows:

$${\delta }^{18}{\rm{O}}={R}_{sample}/{R}_{reference}-1$$

where *R* denotes the ratio of numbers (*N*) of each isotope, as follows:

$$R=N({}^{18}{\rm{O}})/N({}^{16}\,{\rm{O}})$$

The standard deviation of replicates was approximately 0.2‰ (1*σ*) for measurements^13^.

### Bayesian inference of vertical seed dispersal

As explained in the above section, we estimated vertical seed dispersal distances through two processes. First, we estimated the altitude of the mother plant by determining the negative correlation between altitudes and the oxygen isotope ratio of non-dispersed seeds (i.e. calibration line), and using the oxygen isotope ratio of a dispersed seed. Secondly, we estimated vertical seed dispersal distance by subtracting the altitude of a mother plant from that of a dispersed seed. We used the negative correlation between altitudes and the oxygen isotope ratio of *A. arguta* seeds reported in the previous study (*R*^2^ = 0.55, y = −0.0024 × +19.0213)^13^. This study was conducted at Okutama in 2013, and the non-dispersed seeds of *A. arguta* were collected at 600–1,280 m a.s.l. The negative correlation was relatively shallow compared with the other species examined (*R*^2^ > 0.83)^8^. This means that simply using the negative correlation as a calibration line could results in a relatively large estimation error of the altitude identification of a mother plant, and thus, vertical seed dispersal distance. To evaluate these errors quantitatively, we used Bayesian inference to estimate them as derivative parameters. We used the Markov chain Monte Carlo method (MCMC) to characterize the posterior distributions of the parameters. To run the MCMC algorithm for model fitting, we used WinBUGS (ver. 1.4.3^80^). Uniform distributions were used as the prior distribution for all parameters. We ran the MCMC algorithm for three independent chains of 110,000 iterations. In each chain, the first 10,000 iterations were abandoned as a burn-in and the remaining chain of 100,000 iterations was thinned every 50 steps, resulting in 2,000 values per chain sampled from the posterior. Finally, 6,000 samples were obtained from three chains, which were used to yield the posterior distributions and summarize the parameters. The model convergence was assessed visually and using $\hat{R}$ values (the Gelman–Rubin statistic), and the $\,\hat{R}$ of all the parameters was <1.04, indicating that our model convergence was good^81^.

Calibration lines (more specifically, intercepts of them), which are determined by the monthly mean air temperature during seed maturation, can vary annually^8^. Based on this, we checked the monthly mean air temperature at the nearest meteorological station located 2 km from study site (530 m a.s.l.) to determine whether we could use the calibration line in 2013 to estimate the seed dispersal distance of mammal-dispersed seeds collected in 2010 and 2011. The monthly mean air temperatures in July, during *A. arguta* seed maturation, were 23.8, 23.5, and 23.3 °C in 2010, 2011, and 2013, respectively. In other words, there was a 0.5 °C difference between 2010 and 2013, and a 0.2 °C difference between 2011 and 2013. Referring the literature which regarded 0.2 °C difference between years as identical and 0.8 °C difference as different^8^, we excluded dispersed seeds collected in 2010 from our analysis of seed dispersal distance. As a result, we only used dispersed seeds collected in 2011 for seed dispersal distance estimate. Based on the lapse rate (i.e., ca. −0.6 °C per 100 m altitude)^10^, a 0.2 °C difference corresponds to 33.3 m, implying that the oxygen isotope ratio of non-dispersed seeds at some altitude in 2011 might be equal to the one at the 33.3 m higher altitude in 2013. This suggests that we might underestimate the altitude of a mother plant by 33.3 m and thus overestimate seed dispersal distance, but subtracting 33.3 m from estimated seed dispersal distance do not substantially influence our results (see the large negative value of vertical seed dispersal distance in *Results*).

### Relationship between absolute vertical seed dispersal distance and home range of animals

To evaluate the relationship between vertical seed dispersal distance and home range of animals, we first determined the home range of each animal observed based on the literature^19^. We excluded several references reporting home ranges of animals in environments that were very different from ours (e.g. small islands, agricultural lands). For bears, we used their home ranges in Okutama (our study area). Then we averaged the home ranges from the literature for each species. Finally, we conducted a logarithmic regression between the mean vertical seed dispersal distance of *A. arguta* and the home range of each dispersal species^77^.

## Supplementary information

Supporting Information

## References

[1] Urban MC (2015). Accelerating extinction risk from climate change. Science..

[2] Davis MB, Shaw RG (2001). Range Shifts and Adaptive Responses to Quaternary Climate Change. Science..

[3] Lenoir J, Gegout JC, Marquet PA, de Ruffray P, Brisse H (2008). A Significant Upward Shift in Plant Species Optimum Elevation During the 20th Century. Science..

[4] Kelly AE, Goulden ML (2008). Rapid shifts in plant distribution with recent climate change. Proc. Natl. Acad. Sci. USA.

[5] Suzuki SN, Ishihara MI, Hidaka A (2015). Regional-scale directional changes in abundance of tree species along a temperature gradient in Japan. Glob. Chang. Biol..

[6] Matías L, Jump AS (2015). Asymmetric changes of growth and reproductive investment herald altitudinal and latitudinal range shifts of two woody species. Glob. Chang. Biol..

[7] Corlett RT, Westcott DA (2013). Will plant movements keep up with climate change?. Trends Ecol. Evol..

[8] Naoe S (2016). Mountain-climbing bears protect cherry species from global warming through vertical seed dispersal. Curr. Biol..

[9] González-Varo JP, López-Bao JV, Guitián J (2017). Seed dispersers help plants to escape global warming. Oikos.

[10] Barry, R. G. & Chorley, R. J. *Atmosphere, Weather and Climate*. (Routledge, 2009).

[11] Piotti A, Leonardi S, Piovani P, Scalfi M, Menozzi P (2009). Spruce colonization at treeline: where do those seeds come from?. Heredity..

[12] Johnson JS, Gaddis KD, Cairns DM, Krutovsky KV (2017). Seed dispersal at alpine treeline: an assessment of seed movement within the alpine treeline ecotone. Ecosphere.

[13] Naoe S, Tayasu I, Masaki T, Koike S (2016). Negative correlation between altitudes and oxygen isotope ratios of seeds: exploring its applicability to assess vertical seed dispersal. Ecol. Evol..

[14] Driscoll DA (2014). The Trajectory of Dispersal Research in Conservation Biology. Systematic Review. PLoS One.

[15] Morgan JW, Venn SE (2017). Alpine plant species have limited capacity for long-distance seed dispersal. Plant Ecol..

[16] Rötzer T, Chmielewski FM (2001). Phenological maps of Europe. Clim. Res..

[17] Koike S, Kasai S, Yamazaki K, Furubayashi K (2008). Fruit phenology of *Prunus jamasakura* and the feeding habit of the Asiatic black bear as a seed disperser. Ecol. Res..

[18] Hopkins AD (1919). The Bioclimatic Law as Applied to Entomological Research and Farm Practise. Sci. Mon..

[19] Ohdachi, S. D., Ishibashi, Y., Iwasa, M. A. & Saitioh, T. *The Wild mammals of Japan*. (Shoukadoh, 2015).

[20] Boyle WA, Martin K (2015). The conservation value of high elevation habitats to North American migrant birds. Biol. Conserv..

[21] Osborne W, Green K (1992). Seasonal Changes in Composition, Abundance and Foraging Behavior of Birds in the Snowy Mountains. Emu.

[22] Izumiyama S, Shiraishi T (2004). Seasonal changes in elevation and habitat use of the Asiatic black bear (*Ursus thibetanus*) in the Northern Japan Alps. Mammal Study.

[23] Alexander JM, Diez JM, Levine JM (2015). Novel competitors shape species’ responses to climate change. Nature.

[24] Savolainen O, Pyhäjärvi T, Knürr T (2007). Gene Flow and Local Adaptation in Trees. Annu. Rev. Ecol. Evol. Syst..

[25] HilleRisLambers J, Harsch MA, Ettinger AK, Ford KR, Theobald EJ (2013). How will biotic interactions influence climate change-induced range shifts?. Ann. N. Y. Acad. Sci..

[26] Herrera, C. M. Seed dispersal by vertebrates. in *Plant–animal interactions: an evolutionary approach* (eds Herrera, C. M. & Pellmyr, O.) 185–208 (Blackwell Publishing, 2002).

[27] Masaki T (2012). Fleshy fruit characteristics in a temperate deciduous forest of Japan: how unique are they?. J. Plant Res..

[28] Koike S, Morimoto H, Goto Y, Kozakai C, Yamazaki K (2008). Frugivory of carnivores and seed dispersal of fleshy fruits in cool-temperate deciduous forests. Journal of Forest Research.

[29] Fuentes M (1992). Latitudinal and elevational variation in fruiting phenology among western European bird-dispersed plants. Ecography..

[30] Hanya G (2005). Comparisons of dispersal success between the species fruiting prior to and those at the peak of migrant frugivore abundance. Plant Ecol..

[31] Carnicer J, Jordano P, Melián CJ (2009). The temporal dynamics of resource use by frugivorous birds: a network approach. Ecology.

[32] Koike S (2010). Long-term trends in food habits of Asiatic black bears in the Misaka Mountains on the Pacific coast of central Japan. Mamm. Biol..

[33] Tsuji Y, Yasumoto Y, Takatsuki S (2014). Multi-annual variation in the diet composition and frugivory of the Japanese marten (*Martes melampus*) in western Tokyo, central Japan. Acta Theriol..

[34] Koike S (2011). Estimate of the seed shadow created by the Asiatic black bear *Ursus thibetanus* and its characteristics as a seed disperser in Japanese cool-temperate forest. Oikos.

[35] Arimoto, I., Okamura, H., Koike, S., Yamazaki, K. & Kaji, K. Behavior and habitat of Asiatic black bear (*Ursus thibetanus*) inhabiting near settlements. *Mamm. Sci*. **54**, 19–31 (in Japanese with English summary) (2014).

[36] Koike, S. & Masaki, T. Frugivory of carnivora in central and southern parts of Japan analyzed by literature search. *J. Japanese For. Soc*. **90**, 26–35 (in Japanese with English summary) (2008).

[37] Körner, C. & Spehn, E. M. *Mountain Biodiversity: A Global**Assessment*. (Parthenon Publishing, 2002).

[38] Arase, T. & Uchida, T. Regional differences in the fruit morphology and yield of hardy kiwifruit (*Actinidia arguta*) in the central and southern part of Nagano Prefecture. *Bull. Shinshu Univ* **7**, 11–19 (in Japanese with English summary) (2009).

[39] Barthlott W, Mutke J, Rafiqpoor D, Kier G, Kreft H (2005). Global Centers of Vascular Plant Diversity and Holger KREFT (Bonn). *Nov. Acta*. Leopoldina NF.

[40] Fujimori, T. *Ecological and silvicultural strategies for sustainable forest management*. (Elsevier Science, 2001).

[41] Körner, C. *Alpine Plant Life: Functional Plant Ecology of High Mountain Ecosystems*. (Springer, 2003).

[42] Silvertown J, Franco M, Perez-Ishiwara R (2001). Evolution of senescence in iteroparous perennial plants. Evol. Ecol. Res..

[43] Jordano, P. Fruits and frugivory. in *Seeds: the ecology of regeneration in plant communities* (ed. Fenner, M.) 125–166 (CAB International, 2000).

[44] Gómez JM, Schupp EW, Jordano P (2019). Synzoochory: the ecological and evolutionary relevance of a dual interaction. Biol. Rev..

[45] Sone K (2002). Hoarding of acorns by granivorous mice and its role in the population processes of *Pasania edulis* (Makino) Makino. Ecol. Res..

[46] Ida H, Nakagoshi N (1996). Gnawing damage by rodents to the seedlings of *Fagus crenata* and *Quercus mongolica var. grosseserrata* in a temperate Sasa grassland-deciduous forest series in southwestern Japan. Ecol. Res..

[47] Pons J, Pausas JG (2007). Acorn dispersal estimated by radio-tracking. Oecologia.

[48] Gómez JM (2003). Spatial patterns in long-distance dispersal of *Quercus ilex* acorns by jays in a heterogeneous landscape. Ecography..

[49] Vander Wall SB, Balda RP (1977). Coadaptations of the Clark’s Nutcracker and the Pinon Pine for Efficient Seed Harvest and Dispersal. Ecol. Monogr..

[50] Mise Y, Yamazaki K, Soga M, Koike S (2016). Comparing methods of acquiring mammalian endozoochorous seed dispersal distance distributions. Ecol. Res..

[51] Tsuji Y, Morimoto M (2016). Endozoochorous seed dispersal by Japanese macaques (*Macaca fuscata*): Effects of temporal variation in ranging and seed characteristics on seed shadows. Am. J. Primatol..

[52] Tsuji Y, Okumura T, Kitahara M, Jiang Z (2016). Estimated Seed Shadow Generated by Japanese Martens(*Martes melampus*): Comparison with Forest-Dwelling Animals in Japan. Zoolog. Sci..

[53] Andrew Royle J, Rubenstein DR (2004). The role of species abunande in detemining breeding origins of migratory birds with stable isotopes. Ecol. Appl..

[54] Tsuji Y, Sato K, Sato Y (2011). The role of Japanese macaques (Macaca fuscata) as endozoochorous seed dispersers on Kinkazan Island, northern Japan. Mamm. Biol..

[55] Hämäläinen, A. *et al*. The ecological significance of secondary seed dispersal by carnivores. *Ecosphere* **8** (2017).

[56] Tsuji Y, Fujita S, Sugiura H, Saito C, Takatsuki S (2006). Long-term variation in fruiting and the food habits of wild Japanese macaques on Kinkazan Island, northern Japan. Am. J. Primatol..

[57] Takatsuki S, Miyaoka R, Sugaya K (2018). A Comparison of food habits between Japanese marten and raccoon dog in western Tokyo with reference to fruit use. Zoolog. Sci..

[58] Naoe S, Masaki T, Sakai S (2018). Effects of temporal variation in community-level fruit abundance on seed dispersal by birds across woody species. Am. J. Bot..

[59] Boutin S (2006). Anticipatory reproduction and population growth in seed predators. Science..

[60] Kelly D, Koenig WD, Liebhold AM (2008). An intercontinental comparison of the dynamic behavior of mast seeding communities. Popul. Ecol..

[61] Wolff JO (1996). Population Fluctuations of Mast-Eating Rodents Are Correlated with Production of Acorns. J. Mammal..

[62] Mokany K, Prasad S, Westcott DA (2015). Impacts of climate change and management responses in tropical forests depend on complex frugivore-mediated seed dispersal. Glob. Ecol. Biogeogr..

[63] Mclachlan JS, Clark JS, Manos PS (2005). Molecular indicators of tree migration capacity under rapid climate change. Ecology.

[64] Ikeda H (1984). Raccoon dog scent marking by scats and its significance in social behaviour. J. Ethol..

[65] McLellan ML, McLellan BN (2015). Effect of season and high ambient temperature on activity levels and patterns of grizzly bears (*Ursus arctos*). PLoS One.

[66] Spooner, F. E. B., Pearson, R. G. & Freeman, R. Rapid warming is associated with population decline among terrestrial birds and mammals globally. *Global Change Biology*, 10.1111/gcb.14361 (2018). 10.1111/gcb.14361 30033551

[67] Westcott DA (2000). Patterns of movement and seed dispersal of a tropical frugivore. Oecologia.

[68] Jordano P, García C, Godoy JA, García-Castaño JL (2007). Differential contribution of frugivores to complex seed dispersal patterns. Proc. Natl. Acad. Sci. USA.

[69] Japan Meteorologica Agency. Mean monthly meteorological data at Ogouchi between 1981–2010. (2016). Available at: http://www.data.jma.go.jp/obd/stats/etrn/view/nml_amd_ym.php?prec_no=44&block_no=0365&year=&month=&day=&view=) (in Japanese) (Accessed: 22th April 2019).

[70] Ohashi, H., Hoshino, Y. & Oono, K. Long-term changes in the species composition of plant communities caused by the population growth of Sika deer (*Cervus nippon*) in Okutama, Tokyo. *Veg. Sci*. **24**, 123–151 (in Japanese with English summary) (2007).

[71] Maeda, T., Kato, T. & Shimazaki, Y. Studies on forest vegetation of the ‘Chichibu’ mountains district. *J. Japanese For. Soc*. **32**, 340–342 (in Japanese with English summary) (1950).

[72] Maeda, T. & Yoshioka, J. Studies on the Vegetation of Chichibu Mountain Forest (II): The Plant Communities of the Temperate Mountain Zones, with Plates II-III. *Bull. Tokyo Univ. For*. **42**, 129–150 (in Japanese with English summary) (1952).

[73] Horikawa, Y. *Atlas of the Japanese flora an introduction to plant sociology of East Asia*. *Atlas of the Japanese flora an introduction to plant sociology of East Asia* (Gakken, 1972).

[74] Mori H, Kamijo T, Masaki T (2016). Liana distribution and community structure in an old-growth temperate forest: the relative importance of past disturbances, host trees, and microsite characteristics. Plant Ecol..

[75] Yasumoto Y, Takatsuki S (2015). The Japanese marten favors *Actinidia arguta*, a forest edge liane as a directed seed disperser. Zoolog. Sci..

[76] Koike S, Masaki T (2019). Characteristics of fruits consumed by mammalian frugivores in Japanese temperate forest. Ecol. Res..

[77] Biodiversity Center of Japan. Vegetation Survey: 6th and 7th National Survey on the Natural Environment. *Nature Conservation Bureau, Ministry of the Environment, Fujiyoshida, Yamanashi* Available at: http://gis.biodic.go.jp/webgis/?_ga=2.37760675.1886663512.1510272970- 1896608806.1510272970. (Accessed: 22th April 2019) *(in Japanese)* (2006).

[78] Iacumin P, Bernini L, Boschetti T (2009). Climatic factors influencing the isotope composition of Italian olive oils and geographic characterisation. Rapid Commun. Mass Spectrom..

[79] Brand WA (2009). Comprehensive inter-laboratory calibration of reference materials for d^18^O versus VSMOW using various on-line high-temperature conversion techniques. Rapid Commun. mass Spectrom..

[80] Lunn DJ, Thomas A, Best N, Spiegelhalter D (2000). WinBUGS – A Bayesian modelling framework: Concepts, structure, and extensibility. Stat. Comput..

[81] Gelman, A. & Hill, J. *Data Analysis Using Regression and Multilevel/Hierarchical Models*. (Cambridge University Press, 2007).

