# Supplementary material for: Downhill seed dispersal by temperate mammals: a potential threat to plant escape from global warming
Source: Sci Rep. 2019 Oct 17;9:14932. doi: 10.1038/s41598-019-51376-6 (PMC6797773; doi:10.1038/s41598-019-51376-6)
Supplement: Supplementary file 1 — Supporting Information [file 41598_2019_51376_MOESM1_ESM.docx]

**Downhill seed dispersal by temperate mammals: a potential threat to plant escape from global warming**

Shoji Naoe^1,†,*^, Ichiro Tayasu^2,3^, Yoichiro Sakai^3,4^, Takashi Masaki^1^, Kazuki Kobayashi^5^, Akiko Nakajima^5^, Yoshikazu Sato^5,††^, Koji Yamazaki^6,†††^, Hiroki Kiyokawa^7^, Shinsuke Koike^8^

^1^Forestry and Forest Products Research Institute, Matsunosato 1, Tsukuba, Ibaraki 305–8687, Japan; ^2^Research Institute for Humanity and Nature, 457-4 Motoyama, Kamigamo, Kita-ku, Kyoto 603-8047, Japan; ^3^Center for Ecological Research, Kyoto University, Hirano 2-509-3, Otsu, Shiga 520-2113, Japan; ^4^Lake Biwa Environmental Research Institute, 5-34 Yanagasaki, Ohtsu, Shiga 520-0022, Japan; ^5^College of Bioresource Sciences, Nihon University, Fujisawa, Kanagawa 252-8510, Japan; ^6^Ibaraki Nature Museum, 700 Ohsaki, Bando, Ibaraki, 306-0622, Japan; ^7^Laboratory of Biodiversity Science, School of Agriculture and Life Sciences, University of Tokyo. 1-1-1 Yayoi, Bunkyo-ku, Tokyo 113-8656, Japan; ^8^Tokyo University of Agriculture and Technology, 3-5-8 Saiwai, Fuchu, Tokyo, 183-8509, Japan

^†^Present address: Tohoku Research Center, Forestry and Forest Products Research Institute, 92-25 Nabeyashiki, Shimokuriyagawa, Morioka, Iwate 020–0123, Japan; ^††^Present address: Rakuno Gakuen University, 582 Bunkyodai-Midorimachi, Ebetsu, Hokkaido, 069-8501, Japan; ^†††^Present address: Department of Forest Science, Tokyo University of Agriculture, 1-1-1 Sakuragaoka, Setagaya, Tokyo 156-8502, Japan

***Correspondence:** Tel.: +81–19–648–3942, Email: naoeshoji@affrc.go.jp

**Supporting Information**

**Figure S1.** Location of Kanto Mountains (a), topography of Kanto Mountains and the surrounding area (b).

**Figure S2.** Bayesian estimated vertical dispersal distance of each seed by Asian black bear, Japanese macaque, raccoon dog, and Japanese marten. Values are means with 95% credible intervals. Blue and orange circles indicate uphill and downhill seed dispersal whose 95% credible intervals did not include zero, respectively; gray circles indicate seed dispersal whose 95% credible intervals included zero.


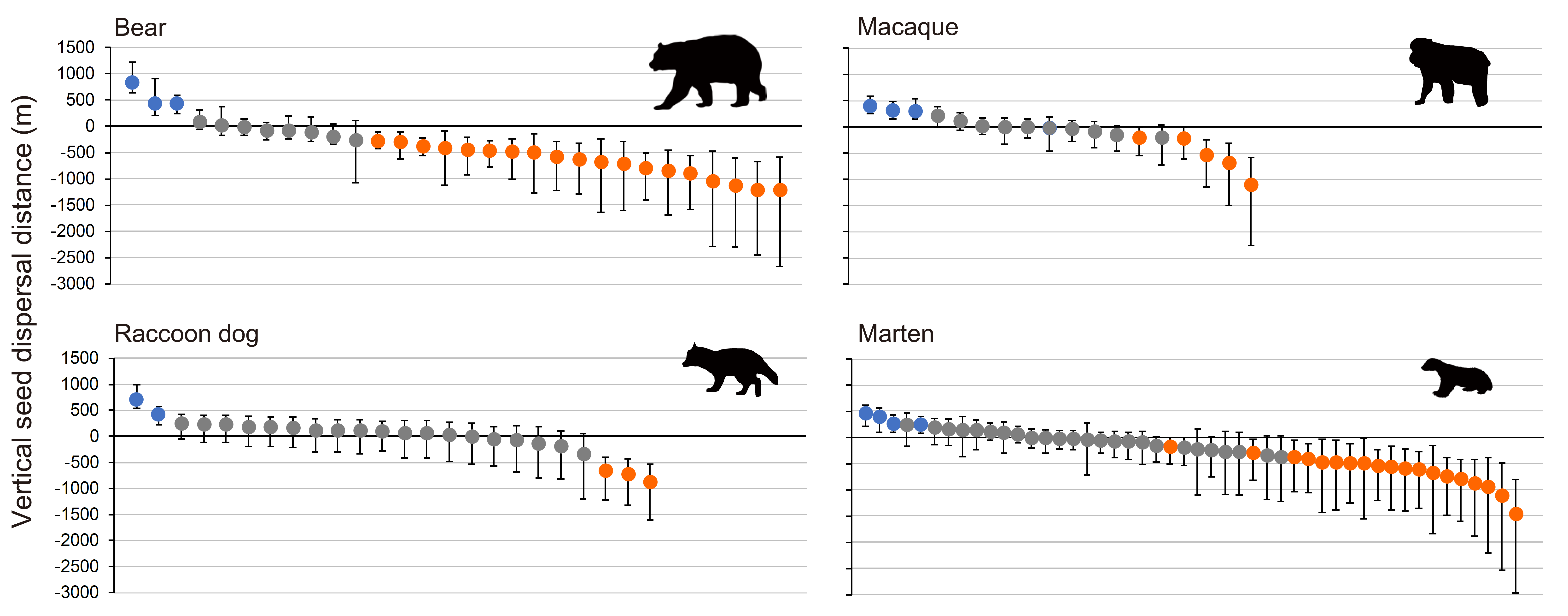


**Figure S3.** Histograms of vertical seed dispersal by Asian black bear, Japanese macaque, raccoon dog, and Japanese marten.

**Table S1.** Ecological characteristics of Asian black bear, Japanese macaque, raccoon dog, and Japanese marten.

^*^Data from Ohdachi *et al.* (2015).

^†^Data from Hamada *et al.* (2003).

^‡^Data from Shibata (1996).

^§^Data from Ohdachi *et al.* (2015) and Suzuki *et al.* (1977).

Ohdachi, S. D., Ishibashi, Y., Iwasa, M. A., & Saitioh, T. (2015). *The Wild mammals of Japan* (2nd ed.). Kyoto: Shoukadoh.

Hamada, Y., Hayakawa, S., Suzuki, J., Watanabe, K., & Ohkura, S. (2003). Seasonal variation in the body fat of Japanese macaques *Macaca fuscata*. *Mammal Study*, 28, 79–88. doi: 10.3106/mammalstudy.28.79

Shibata, F. 1996. Raccoon dog. In T. Kawamichi (Ed.), *The Encyclopaedia of Animals in Japan Vol. 1: Mammals 1* (pp., 116–119). Tokyo: Heibonsha (in Japanese).

Suzuki, S., Miyao, T.., Nishizawa, T., Takada, Y.. (1977). Studies on Mammals of the Mt. Kiso-Komagatake, Central Japan Alps III: food Habit of the Japanese marten in upper part of low mountainous zone and the sub-alpine zone of the Mt. Kiso-Komagatake. *Journal of the Faculty of Agriculture Shinshu University, 14,* 147–177 (in Japanese).
